# Monkeypox: A comprehensive review of a multifaceted virus

**DOI:** 10.1016/j.imj.2023.04.009

**Published:** 2023-05-12

**Authors:** Randa Elsheikh, Abdelrahman M. Makram, Tamilarasy Vasanthakumaran, Shubham Tomar, Khizer Shamim, Nguyen Dong Tranh, Sara S. Elsheikh, Nguyen Thanh Van, Nguyen Tien Huy

**Affiliations:** a Deanery of Biomedical Sciences at Edinburgh Medical School, University of Edinburgh, Edinburgh EH10 5HF, United Kingdom; b Online Research Club (http://www.onlineresearchclub.org), Nagasaki 852-8523, Japan; c School of Public Health, Imperial College London, London SW7 2BX, United Kingdom; d Global Clinical Scholars Research Training, Harvard Medical School, Boston, MA 02115, USA; e Tata Memorial Hospital, Varanasi 400012, India; f DataQ Health, Karachi 75100, Pakistan; g Department of Infection Control, Binh Dinh Provincial General Hospital, Binh Dinh 55000, Vietnam; h Faculty of Medicine, Zagazig University, Zagazig 44519, Egypt; i School of Tropical Medicine and Global Health, Nagasaki University, Nagasaki 852-8523, Japan

**Keywords:** Monkeypox, Orthopoxvirus, Outbreak, Smallpox

## Abstract

In May 2022, the world witnessed the re-emergence of the zoonotic disease monkeypox. While this was not the first epidemic of this disease, what differentiated the outbreak was the rapid global spread and increase of cases, which led the WHO to declare monkeypox a global health emergency. Although the disease spreads mainly through inadequately cooked meat of various rodent species, this virus also shows droplet, respiratory, sexual, and even vertical transmission. Monkeypox further multiplies in lymphoproliferative organs and presents with a classical smallpox-like rash, fever, headache, and muscle aches. Diagnosis is confirmed with a polymerase-chain-reaction test and is managed largely supportively with possible usage of some antivirals and immunoglobulins. Moreover, some pre-exposure and postexposure prophylactic vaccines have been developed. This paper aims to conduct an in-depth review of the historical epidemics, transmission, pathophysiology, clinical presentation, and management of the monkeypox disease.

## Highlights

• The world has experienced an unprecedented monkeypox outbreak that started in known endemic countries and spread to nonendemic countries.
• The recent outbreak might be an indicator of new viral mutations.
• The decrease in smallpox vaccination following its eradication is hypothesized to have contributed to the recent surge in cases.
• Mass vaccination using smallpox or monkeypox vaccines may not be efficient. Treating this viral outbreak like COVID-19 should be discouraged.

## Graphical abstract

**Figure:**
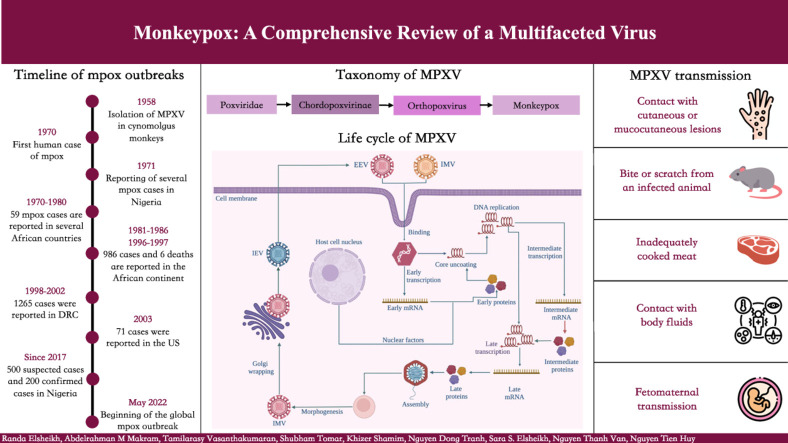

## 1 Introduction

On the 7th of May 2022, the first case of monkeypox (known now as mpox) was detected in the United Kingdom (UK) in a traveler returning from Nigeria. Although an outbreak was ongoing in Nigeria and other African countries since 2017, the disease seemed to be limited to previously endemic areas [1].

**Fig. 1:**
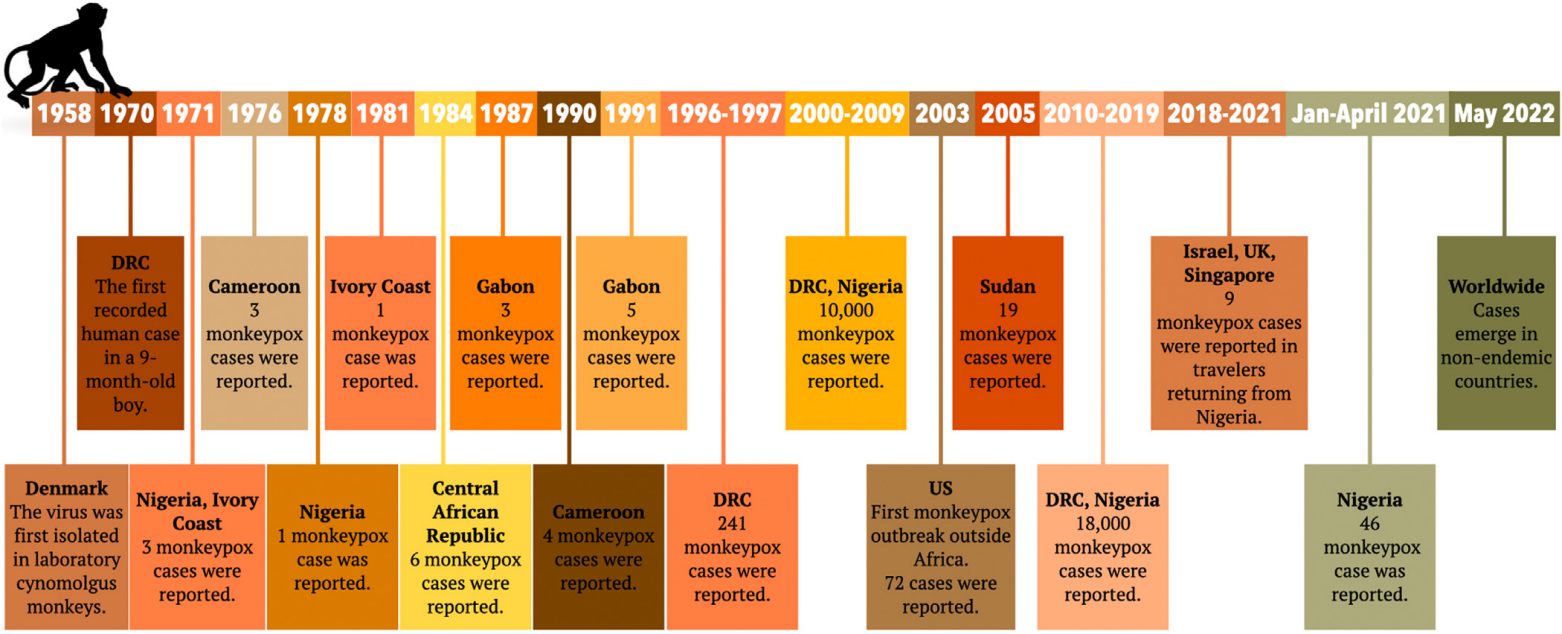
Timeline of monkeypox outbreaks [3],[4].

However, following the virus's first detection in the UK, several countries have reported new cases and, as of the 22nd of September (according to the US Centers for Disease Control and Prevention (CDC)), approximately 65,415 cases and 26 deaths have been registered in 106 countries, 99 of which had no previously historically registered cases of the disease (Fig. 1) [2]. Despite the mild clinical picture of the virus, what alarmed the global community was the high, unexpected human-to-human transmission rate the virus exhibited during the outbreak [5], which suggested the emergence of a mutant strain. On the 23rd of July, monkeypox was declared a global health emergency [6].

Monkeypox is a zoonotic disease caused by the monkeypox virus (MPXV), a double-stranded DNA virus belonging to the *Orthopoxvirus* genus, the same genus as smallpox with whom it shares some similarities, despite being less severe. Although scientists first identified the virus in laboratory monkeys in 1958, the first-ever human infection was documented in 1970 in the Democratic Republic of Congo [7]. Two clades of the virus have been identified: the West African and the Congo Basin clades. The former was found to cause milder disease than the latter with a case fatality rate of 3.6% and 10.6%, respectively [8]. The detection of the virus was concomitant with the vaccination campaigns for the eradication of smallpox, whose vaccine has proved to provide cross-protection against monkeypox. In their systematic review, Bunge et al. argued that the recent increase in the frequency of monkeypox outbreaks might be attributable to the decreased vaccination coverage against smallpox in the new generations following the eradication of the virus [9]. Although vaccination of the general population was not advised during the most recent outbreak, the WHO recommended the administration of postexposure preventive vaccination for contacts as well as primary preventive vaccination for high-risk groups like men who have sex with men (MSM), in whom the disease shows a predominance, individuals with multiple sexual partners, healthcare workers, children, and pregnant women [1]. Owing to the documented high effectiveness of the smallpox vaccine against monkeypox, it was also offered to nonimmunocompromised adults [10].

While the literature has covered different aspects of monkeypox since the onset of the outbreak, a review that addresses the disease in a comprehensive way is lacking. This article aims at serving as an integrative, comprehensive review of the most recent monkeypox outbreak, providing a detailed insight into the genomics of the causative virus, the historical background of previous outbreaks, and highlighting the available treatment and prevention measures as well as the principal public health strategies implemented to control the spread of the disease.

## 2 Monkeypox epidemics in the past

Monkeypox virus was first isolated in 1958 in Copenhagen from a group of laboratory cynomolgus monkeys used for researching poliovirus vaccines after one of the monkeys had developed pox-like skin cutaneous lesions [11]. The virus was not regarded as a threat due to the concomitant vaccinia vaccine campaign against smallpox, which was shown to provide cross-protection against monkeypox. This campaign ended in 1981 when the World Health Organization (WHO) officially declared the complete eradication of smallpox [12].

The first human infection with MPXV was documented in 1970 in a 9-month-old boy in the Democratic Republic of Congo (DRC), previously known as Zaire [13]. Following that, several monkeypox cases were reported in 1971 in Nigeria, the most populous African country, one of which was a 4-year-old who had not previously received any smallpox vaccination. In 1978, an additional case was confirmed, after which no cases were reported in the country until 2017 [14]. A total of 59 cases are said to have been documented in Cameroon, Côte d'Ivoire, Liberia, Nigeria, Sierra Leone, and DRC between 1970 and 1980. Following these outbreaks, an investigation was requested by the Global Commission for the Certification of Smallpox Eradication to determine the public health burden of monkeypox, which revealed that the infection rates in patients without a smallpox vaccination scar were 8 times higher than in those who had received the vaccine [12].

Further outbreaks were reported in Africa in the years 1981–1986 and 1996–1997, characterized by a lower case-fatality ratio and a greater attack rate than typical outbreaks. A total of 986 cases and 6 deaths were recorded in the continent during that period which was considered the largest outbreak since virus isolation [15]. Nevertheless, the mutation of the virus into a more virulent form was excluded by comparing the strains of different outbreaks [15]. The rate of infections continued to rise in DRC between 1998 and 2002 reaching 1,265 infections. This number, however, is thought to be much lower as the number of collected specimens was only 215, and only a small proportion was revealed to be positive for monkeypox [16].

In 2003, the virus made its first appearance outside of Africa in the United States. A total of 71 cases were reported by the CDC in Wisconsin (39), Indiana (16), Illinois (12), Missouri (2), Kansas (1), and Ohio (1) [17]. The source of infection was confirmed to be prairie dogs that were exposed to Gambian pouched rats and dormice imported from Ghana [13].

In 2005, concerns were raised following reporting of a case in southern Sudan, which represented the first African case outside the Congo Basin and western African regions [18].

In September 2017, Nigeria witnessed a re-emergence of the disease after almost 4 decades of latency [14]. According to the WHO, since 2017, more than 500 suspected cases and more than 200 confirmed cases have been reported in Nigeria, with a case-fatality ratio of approximately 3% [13]. From 2018 to 2021, several cases were reported in Israel, the UK, and Singapore in returning travelers from Nigeria [3].

May 2022 registered a surge in cases in nonendemic areas of the world, which prompted the global healthcare authorities to take necessary action to contain the spread of the virus [8]. Fig. 1 summarizes the timeline of all monkeypox outbreaks.

## 3 Virology

**Fig. 2:**
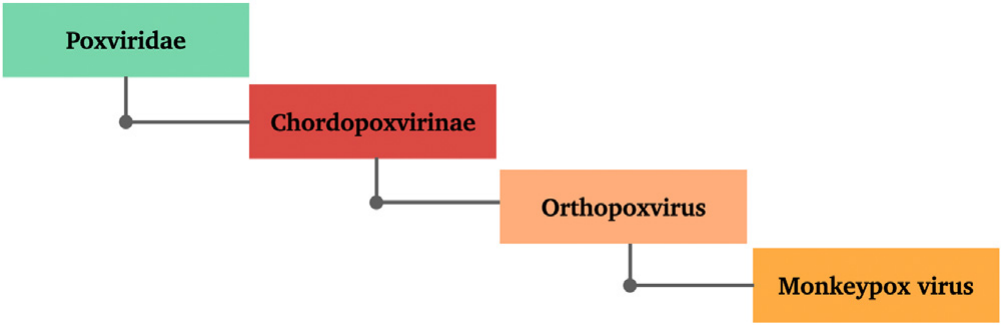
Taxonomy of monkeypox virus.

MPXV is an enveloped, pleomorphic double-stranded DNA virus with a dumbbell-shaped core and lateral bodies belonging to the *Orthopoxvirus* genus, *Poxviridae* family, and *Chordopoxvirinae* subfamily (Fig. 2). Other members of the same genus include smallpox, cowpox, camelpox, ectromelia (mousepox), and vaccinia viruses. The virus shows similar morphology to other members of the same genus, being approximately 140–260 nm in diameter and 220–450 nm in length [16], [19], [20].

### 3.1 Life cycle and replication in host cells

**Fig. 3:**
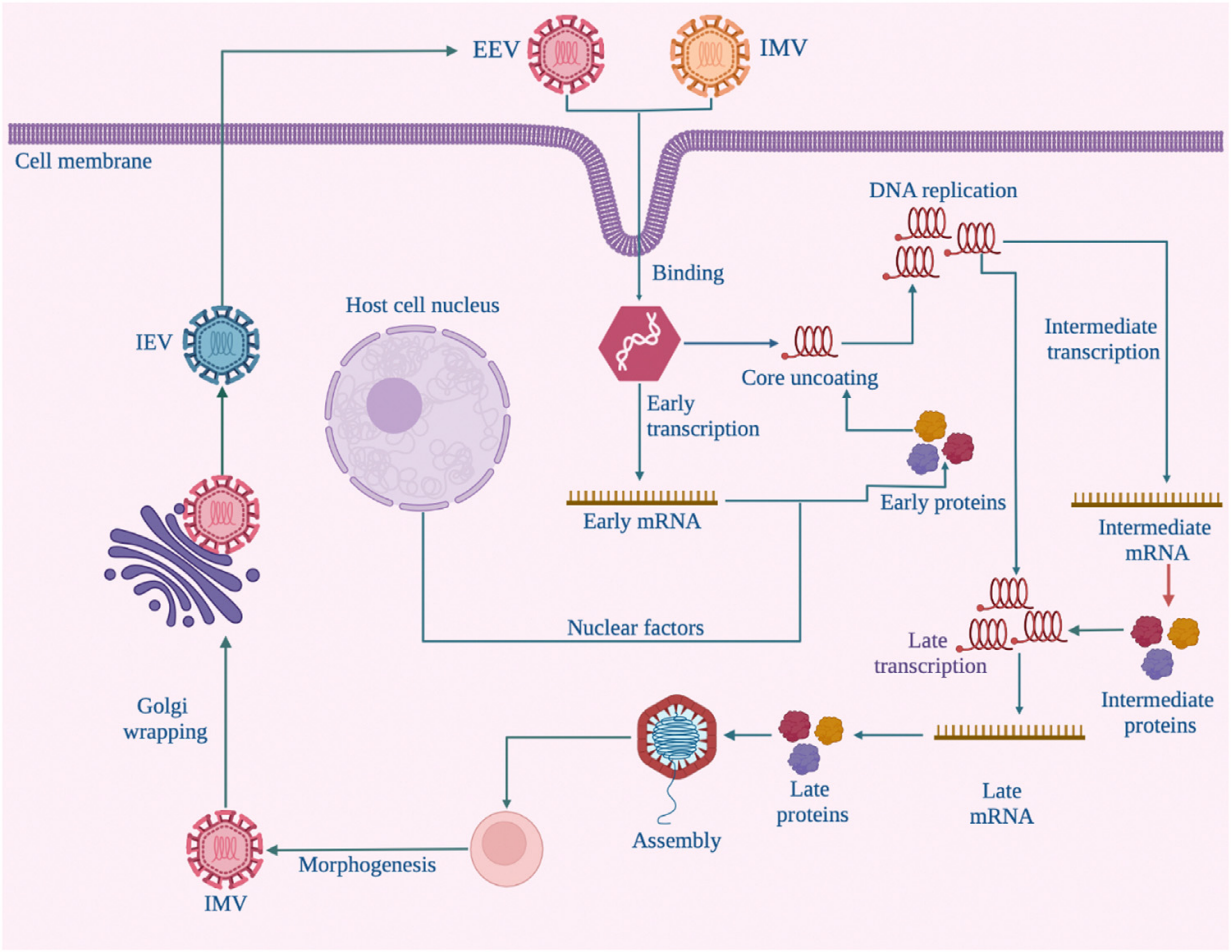
Monkeypox replication in host cells [21]. Abbreviations: EEV, extracellular enveloped virus; IMV, intracellular mature virus; IEV, intracellular enveloped virus.

The replication cycle of MPXV starts with the binding of the infectious viral particles intracellular mature virus (IMV) and extracellular enveloped virus (EEV) to the surface of the target cell. VV–A28, one of the particles’ virion proteins, mediates the virus fusion and release of its core structure into the cytoplasm of the infected cell. The first pathway of early gene expression that leads to viral mRNA synthesis begins under endogenous RNA polymerase influence. The start of the intermediate and late transcription phases is marked by the release of viral DNA into the host's cytoplasm to act as a template for viral replication through a process known as uncoating. This is followed by the assembly of virion particles to form IMV which leads to the formation of intracellular enveloped virus (IEV) which fuses with the cell membrane to form the cell-associated enveloped virus (CEV). While CEV and EEV are thought to be implicated in early cell-to-cell spread, IMV aids viral spread at later stages [21] (Fig. 3).

### 3.2 Pathogenesis

**Fig. 4:**
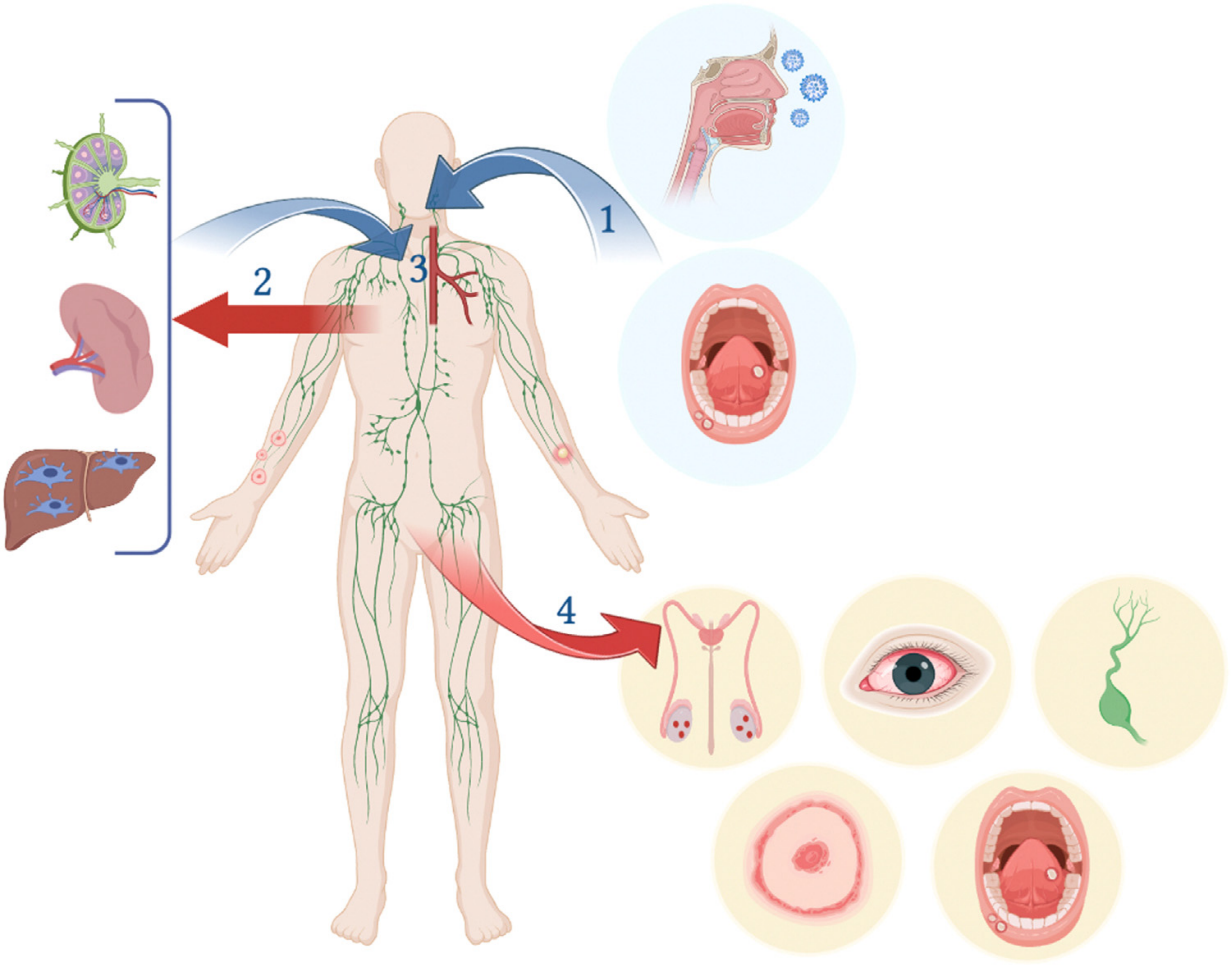
Pathogenesis of monkeypox virus [23], [24]. (1) Monkeypox enters the body via the respiratory or oral route. (2) The virus circulates to the draining lymph nodes. This is followed by primary viremia and dissemination of the virus to distant lymph nodes and secondary lymphoid organs, like the spleen and Kupffer cells of the liver, where viral replication takes place. (3) The virus enters the systemic circulation through lymphoproliferative organs leading to secondary viremia. (4) The virus spreads to distant organs leading to the appearance of lesions on the skin and mucous membranes, lymphadenopathy, and ophthalmic manifestations.

After entering the human body via the airways, the oral cavity, or infected body fluids ingestion, the virus travels to a local lymph node or through the blood, causing primary viremia. It then travels to distant lymph nodes causing lymphadenopathy by enhancing the proliferation of natural killer cells and entering the systemic circulation through the lymphoproliferative organs, causing secondary viremia [22]. Secondary viremia allows viral spread to the lungs, skin, gonads, kidneys, and other distant organs. The spread of the virus to upper skin layers, aided by infected Langerhans cells, leads to the appearance of infective skin lesions. Similarly, migration to the oropharyngeal mucosa leads to the appearance of similar lesions which release infective virions in the saliva upon eruption. Dissemination of the virus to the reproductive organs has also led to its detection in semen, which was correlated to its ability to transmit sexually. Moreover, the virus has been detected in the anorectal region and fecal matter, which is thought to be triggered by the disruption of the anal mucosa seen in MSM leading to the stimulation of regional immunity [23] (Fig. 4).

### 3.3 Viral tropism

Poxviruses exhibit 3 types of viral tropism which play an important role in determining the ability of the virus to infect various host species. The first type, cellular tropism, indicates the different modalities in which the virus replicates in infected species. The second type, known as tissue tropism, refers to the increased viral replication in particular hosts or organs mediated by specific antiviral responses, whereas the third type, known as organism tropism, refers to the pathogenesis and clinical picture of the virus under the effects of host's immune responses. The combination of the last 2 types of tropism determines the host-to-host transmission of the virus [21].

### 3.4 Viral kinetics

Although recent instances of MPXV infection were all phylogenetically connected to the West African clade, at least 2 distinct clades have been found, each with a different fatality rate [25]. According to a study, the terminal areas that encode for expected host-response modifier proteins are where the 2 clades' greatest variety is found [16]. The MPXV homolog of the smallpox inhibitor of complement enzymes (SPICE), which is responsible for the differential in virulence and transmissibility between the Congo Basin and West-African clades, is a potential gene [16,^26^,27].

The Congo Basin isolates have the monkeypox homolog of SPICE (monkeypox inhibitor of complement enzymes (MOPICE)), while the sequenced West African viruses do not [16,[26], [27], [28]. The absence of MOPICE in the West-African MPXV isolates may increase the vulnerability of virion or virus-infected cells to host-derived complement-mediated lysis, resulting in a lower viremia and less severe illness, less seeding of the respiratory mucosa, and less transmissibility [16]. One of the 4 complement regulatory domains seen in SPICE and other *Orthopoxvirus* orthologs is lost in MOPICE from Congo Basin isolates due to a C-terminal truncation [16,^29^,30]. Studies have also shown that the MOPICE protein has complement-inhibiting action despite this truncation [16,^30^,31]; however, the activity is much lower than that described for SPICE [16,31].

Members of the SPICE family are candidates for virulence and transmissibility factors linked to human orthopoxvirus disease due to the presence of MOPICE in the more virulent and transmissible Congo Basin MPXV isolates and SPICE in variola, as well as the absence of MOPICE in the less virulent West-African monkeypox isolates. Candidate open reading frames D10L (host range), B10R (virulence factor for myxoma virus), B14R (interleukin (IL)-1 binding protein), and B19R (serine protease inhibitor-1) are further candidates that may be significant for the virulence and transmissibility of Congo Basin MPXV isolates [16,^26^,27].

### 3.5 Immune evasion

Numerous mechanisms have been developed by orthopoxviruses in general and monkeypox in specific to evade the host's immune response [32]. Among these is the mechanism by which monkeypox prevents cellular signaling by expressing A47, B13, P1, C6, and D11 proteins inhibiting the immune response stimulated by pattern recognition receptors (PPRs) present in the host's cells. Similarly, the virus encodes the F3 protein which can bind to viral double-stranded RNA making the virus unrecognizable by the host's PPRs. Moreover, MPXV encodes the B12R gene, which inhibits the host's regulation of apoptosis, and tumor necrosis factor receptor CrmB, which binds to tumor necrosis factors. MPXV's D14L gene present in the Central African clade secretes MOPICE which prevents the host's immune response by preventing complement activation [21]. Furthermore, MPXV inhibits the activation of CD4^+^ and CD8^+^ T cells, therefore, avoiding the host's systemic immune suppression [32].

### 3.6 Genomic mutation

The West African clade of MPXV is believed to have been present for the past 600 years, and it is an important member of the *Orthopoxvirus* genus that is known to infect humans and animals [33]. This genus has faced a lot of mutations for it to adapt to the different environments in the old and new world, widening the number of hosts it can infect and survive upon [34], [35], [36].

The recent 2022 MPXV outbreak is no exception to that, except that it has gone under the radar for long enough in low-and-middle-income countries. In a study performed by João Paulo Gomes et al., the genomes of the MPXV in 15 infected individuals during the 2022 outbreak were compared. The patients had distinct strains of monkeypox, which the researchers were able to link to an earlier outbreak of the virus that occurred in 2018–2019 in the United Kingdom, Israel, and Singapore and had its origins in Nigeria [37,38]. The testing revealed that the virus had undergone 50 mutations during the previous outbreak in 2018 – up to 12 times more than they had anticipated.

According to another study by the UK Health Security Agency (UKHSA), in comparison to the UK MPXV from 2018, the strain of the 2022 outbreak had 48 unique changes in its genome. Twenty-seven of these mutations were silent, meaning they did not affect the viral proteins while 21 of them altered viral proteins in some way [39].

Based on the known function of the viral proteins, mutations were divided into 3 categories by the UKHSA assessment: low, medium, and high priority for inquiry (Table 1). These evaluations are based on the outcome of complete protein deletion from other orthopoxviruses. Moreover, although epidemic clade mutations are found throughout the genome, there is a limited subset of mutations in proteins that are connected to virus virulence, transmission, or medication interactions. Further research on exact mutations is required to fully understand the genome and pathogenesis of the virus [23]. Table 1 summarizes the main mutations that happened to MPXV that might have led to several outbreaks worldwide.

**Table 1:** The categories of the mutations that happened to the MPXV that is causing the 2022 outbreak in multiple countries.

Two low-priority mutations	Four medium-priority mutations	Three high-priority mutations
• ***C9L(R48C):*** When the interferon-stimulated gene product antagonist is removed from the vaccinia virus, the virus multiplies more poorly. • ***A46L(H221Y):*** Deletion of the vaccinia virus gene decreases the virus's pathogenicity in mice.	• Deletion of the gene from the rabbitpox virus known as ***C23L(S105L)*** – a chemokine-binding protein – increases the severity of the disease in rabbits. • ***C22L(S54F):*** The deletion of a gene from the ectromelia virus that is comparable to the tumor necrosis factor (TNF) receptor-like protein enhances lung disease in mice. • Ankyrin repeat protein, ***C19L(D266N)*** – unknown function – might be host range or virulence. • ***F13L(E353K):*** The target of the tecovirimat antiviral drug – One mutation at F13L has been shown to confer tecovirimat resistance	***Three mutations in the protein B21/B22 (D209N, P722S, M1741I):*** The knock-in of this protein into nonvirulent cowpox strains increased disease severity and mortality in rats. The T-cell inhibitor is also present in the cowpox, camelpox, and horsepox viruses.

### 3.7 Evolution

Monkeypox viral evolution is convoluted. The virus is a member of the family *Poxviridae* and the genus *Orthopoxvirus*. According to comparative genomics, all current *orthopoxvirus* species originated from a single ancestor through a process that was characterized by the loss of a group of accessory genes that was specific to each lineage. The original virus is thought to resemble specific cowpox virus strains, which have a wide range of hosts and almost the entire collection of accessory genes [40], [41], [42]. A comparison of the DNA sequences of other orthopoxviruses showed that the cowpox virus yielded a more comprehensive genomic sequence than other viruses in the same genus [43].

The variola (smallpox) virus and the MPXV are closely related, and both affect people with a febrile rash sickness that is like but less severe than smallpox. According to a study published by Douglass and Dumbell, despite having several deletions, a sequence in the DNA of the MPXV exists that is a homolog of a 1,065-bp open reading frame in the conserved area of the variola virus genome [44]. This bolsters belief in the long-term effectiveness of smallpox eradication and provides compelling evidence that the MPXV is unrelated to the variola virus [25,44]. This is contrary to the belief that the MPXV is a direct descendant of the famed variola virus.

The evolution of zoonotic infections to become more contagious or virulent in humans is a huge concern. An in-depth analysis published by Isidro et al. strongly suggests an ongoing evolution of the MPXV. It used Shotgun metagenomics to quickly reconstruct and characterize the first MPXV outbreak genome sequences, demonstrating that this current MPXV belongs to clade 3 and that the outbreak most likely originated from a single source. Despite clustering with cases from 2018–2019 that were connected to a nation where the virus is endemic, 2022 MPXV (lineage B.1) segregates in a divergent phylogenetic branch, most likely suggesting ongoing accelerated evolution. A comprehensive examination of mutations points to the role played by the host APOBEC3 in viral evolution as well as possible MPXV human adaptations in continuing microevolution. The findings also suggest that genome sequencing might be able to help track the transmission and spread of this double-stranded DNA virus [38].

Moreover, MPXV is likely to evolve slowly as its DNA genome replicates by a viral DNA polymerase with 3′–5′ exonuclease proofreading activity, which has a lower mutation rate than RNA viruses [41,45]. According to molecular clock studies, poxviruses have substitution rates that vary from 2,106 to 1,105 nucleotide substitutions per site each year [41,^46^,47], which might lead to as many as 2 nucleotide modifications in the genome annually which is very slow.

All the above investigations suggest that further studies are required to establish the origin, evolution, and ongoing development of the MPXV.

### 3.8 Genomic difference between MPXV and variola virus

Monkeypox has been long thought to have evolved from smallpox (Table 2). Genetic analysis of the viruses, however, revealed various genomic differences between the 2 viruses. While the 2 organisms carry almost identical central genomic regions encoding some essential functions of the virus (96.3% similarity), terminal regions have been found to encode different proteins which might be responsible for their differences in virulence. The variola virus encodes 2 genes responsible for interferon (IFN) resistance that are not present in monkeypox: C3L, and the long form of E3L. The absence of these genes in monkeypox and a defect in their expression in variola is associated with a weaker person-to-person spread of the viruses. Contrastingly, monkeypox encodes a *β*-binding protein (IL-1) that is considered responsible for the attenuation of the disease caused by the virus [43].

**Table 2:** Major differences between monkeypox and smallpox [3],[20].

	Monkeypox	Smallpox
Causative organism	Monkeypox virus	Variola virus
*Strains*	Congo Basin and West African clades	4 variola major subtypes
*Reservoirs*	Monkeys and rodents	Humans
*Incubation period*	5–21 days	7–19 days
*Prodromal period*	1–4 days	1–4 days
*Duration of illness*	2–4 weeks	Up to 5 weeks
*Period from rash appearance to desquamation*	14–28 days	14–28 days
*Fever*	Low-to-high grade	High grade
*Lymphadenopathy*	Yes	No
*Lesion distribution*	Centrifugal	Centrifugal
*Fatality*	Low	High
*Vaccine*	JYNNEOS and ACAM2000	ACAM2000 and JYNNEOS (approved for prevention against smallpox)

## 4 Transmission

**Fig. 5:**
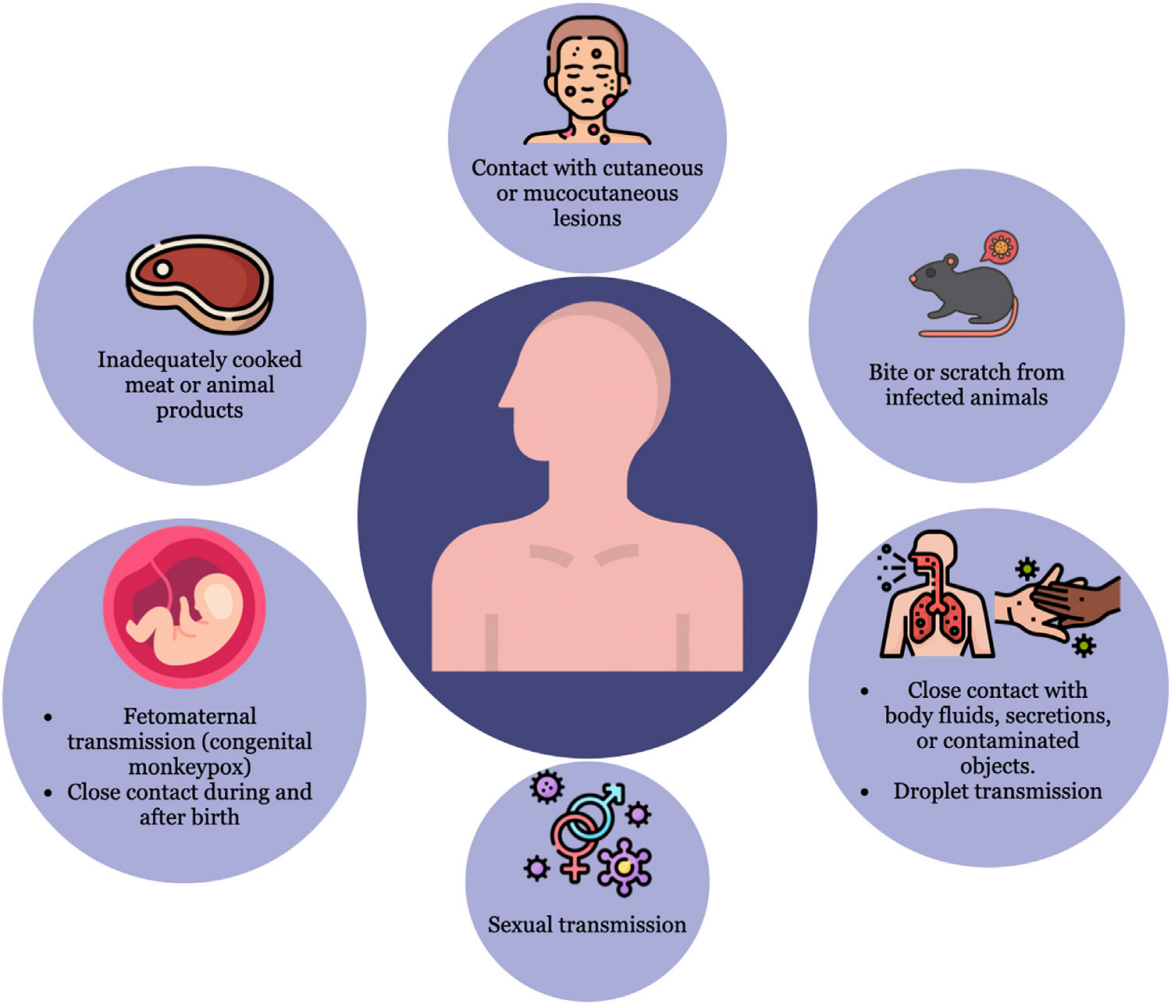
Modes of transmission of monkeypox.

The identified mode of transmission of MPXV in Africa is typically a zoonosis involving living in forested or deforested areas, handling or consuming products of infected meats, and contacting animals or their bodily fluids [4]. Evidence of MPXV infection has been found in many animals in Africa, including rope squirrels, tree squirrels, Gambian pouched rats, dormice, various monkey species, and others. The natural reservoir of monkeypox has not yet been identified, although rodents are the most likely. People living in or near forested areas may have indirect or low-level exposure to infected animals. Eating inadequately cooked meat and other products of infected animals is a possible risk factor. Human-to-human transmission can be caused by close contact with respiratory secretions, skin lesions of an infected person, or recently contaminated objects; however, sexual transmission remains uncertain [13] (Fig. 5).

### 4.1 Respiratory secretions/droplets transmission

The WHO has confirmed that MPXV spreads from human to human, mainly through close contact with respiratory secretions of infected people, skin lesions, or objects contaminated with bodily fluids or diseased tissue of infected people, and through respiratory droplets [48]. In the 2003 US MPXV outbreak, African rodents on a shipment were tested and confirmed the presence of MPXV by polymerase chain reaction (PCR) and virus isolation by the CDC. However, no infected human had contacted these animals; most had direct contact with native prairie dogs, which had closely got the imported African rodents. In addition, it was also reported that a human female became infected with MPXV after a prairie dog was taken into her home; fomites or aerosols were the suspected exposure routes [16]. The MPXV outbreak in the US among humans and captive prairie dogs in 2003 and tracing studies identified the wild African rodents as the probable source [49].

Forty-seven infected patients from the prairie dogs demonstrated high susceptibility of prairie dogs to MPXV [50]. The Discovery of MPXV in the lungs of prairie dogs was the first suggestion that transmission may have occurred via infective droplets [49]. It is now confirmed that MPXV spreads via exhaled large droplets [16]; although they cannot travel a long distance, thus prolonged close contact is required for human-to-human transmission [51,52].

## 5 Sexual transmission

With increasing travel worldwide and the long incubation period, it is unsurprising that MPXV cases have been reported in nonendemic nations. The recent monkeypox outbreak which affected many MSM raised concerns about possible sexual transmission [53]; however, it remains uncertain [13]. Most of the reported cases by the European CDC (ECDC) in the European Union/European Economic Area (EU/EEA) were in young men unrelated to travel, self-identifying as MSM [5].

A group of researchers further investigated sexual transmission and concluded that MPXV can be transmitted through bodily fluids and close skin-to-skin contact from active lesions. The clinical manifestation is generally described as mild, with most lesions on the genitalia or peri-genital areas, indicating that transmission likely occurred during close physical contact during sexual activities. These were believed to be the first cases of MPXV infection with documented transmission through sex [54]. Similar studies from many countries have supported this claim [55], [56], [57], [58]. Therefore, we believe that MPXV can be transmitted sexually, either through close bodily contact or a coitus-related exchange of fluids.

### 5.1 Vertical transmission

Possible vertical transmission was hypothesized by many researchers before. There were also cases of vertical transmission that led to pregnancy loss due to fetal infection [59,60]. This can be attributed to the change in normal immunology physiology in pregnant women, where the cell-mediated effect of T-helper 1 cells is partially inhibited, leading to decreased interferon production and ultimately, a decreased immune response against viral infections [61]. This comes in favor of MPXV as it expresses interferon-alpha/beta binding proteins that can avert the neutralizing effect of the already-weakened interferons [62].

In previous outbreaks, we had 4 cases of reported MPXV infection during pregnancy in the Democratic Republic of Congo (DRC), where one patient had mild disease and no subsequent pregnancy-related complications. However, 2 and one of the 3 other women had a first and second-trimester pregnancy loss, respectively. The second-trimester fetus had apparent hydrops, hepatomegaly, vesicular rash, and confirmed MPXV in its blood using PCR [60]. One more case was reported in another study with the same MPXV features in a 30-week-old fetus [59]. Till now, we are not sure how MPXV can reach the fetus. However, some researchers suggested the hematogenous route [63,64], infecting syncytial- and cytotrophoblastic cells [65,66]. This phenomenon is not new in orthopoxviruses as it was reported in cowpox as well [67], [68], [69].

Therefore, it is important to consider close fetal monitoring in pregnant women with confirmed MPXV infection. Delivery with cesarean section can be an option [70], but further assessment is required before reaching a valid conclusion.

### 5.2 Potential reservoir

Evidence of MPXV infection has been found in various animal species in Africa, including rope squirrels, tree squirrels, Gambian pouched rats, dormice, nonhuman primates, and other species [20,^50^,^71^,72]. Moreover, native prairie dogs, which had close contact with the imported African rodents, were also potential hosts [16]. In 2012, MPXV was isolated in the dead sooty mangabey monkey in the Ivory Coast [73]. This discovery is relatively new and may help to identify the reservoir host. However, the natural reservoir of monkeypox has not yet been identified, although rodents are the most likely [13].

The adaption of *Poxviridae* to new animal hosts has occurred previously. The 2003 US MPXV outbreak added to the breadth of host species capable of supporting MPXV replication and paid attention to the potential for MPXV to expand its geographic range [16]. Furthermore, animal habitat changes, some caused by agricultural expansion, climate change, and urbanization, can increase human exposure to a more significant number and diversity of the small animal population, which may support pathogens [16].

Under laboratory or captive conditions, a broad range of mammalian taxa was likely susceptible to MPXV infection [74], including rabbits, anteaters, opossums, and additional rodent species such as prairie dogs and ground squirrels. It is noted that ground squirrels are highly susceptible to MPXV due to the abundant grassland in Northern America [75]. It is assumed that the maintenance of MPXV in nature may depend on its ability to utilize multiple host species [74]. Also, MPXV can infect a taxonomically wide variety of mammalian species; however, the virus has only been isolated once from a wild animal, a Funisciurus squirrel, in DRC [20],[76]. The extent of viral circulation in animal populations and the precise species that may harbor the virus is not entirely known, although several lines of evidence point to rodents as a potential reservoir [77].

### 5.3 Symptoms and signs

Symptoms of monkeypox resemble those of smallpox in humans and begin with fever, headache, muscle aches, and exhaustion (Table 3). The incubation period of the disease (time from the infection to the appearance of the symptoms) is usually 6 to 13 days but can range from 5 to 21 days [78,79]. The CDC has also estimated that the incubation period between the time of exposure to the onset of the rash was 8.7 days (95% CI: 6.9−11.7) [80].

**Table 3:** Potential supportive care options that can ease the symptoms of monkeypox.

Symptom	Supportive treatment
Respiratory distress	Oral or intravenous antibiotics, nebulizers, and noninvasive ventilation.
Sepsis	Oral or intravenous antibiotics, supplemental oxygen, and corticosteroids.
Mucocutaneous ulcers	Oral or topical analgesics, oral or intravenous antiemetics and antidiarrheal drugs, and oral or intravenous rehydration therapy.
Fever	Antipyretics, external cooling.
Skin lesions	Proper disinfection, moisturized dressings, topical antibiotics, surgical debridement, and skin grafts in severe lesions.
Skin superinfection	Oral or intravenous antibiotics, incision and drainage, advanced wound management (e.g., negative pressure wound therapy).
Inflammation or lymphadenopathy	Oral or intravenous anti-inflammatory drugs and analgesics.
Ocular affection	Ophthalmic antibiotics or antivirals and corticosteroids.

Infection can be divided into 2 phases: the initial invasion period (lasting from 0–5 days) which manifests as high fever, intense headache, lymphadenopathy, back pain, myalgia, and exhaustion, and the skin rash period (appearing within 1−3 days from the onset of fever). The rash occurs more on the face and extremities but can also involve the oral mucous membranes, genitalia, conjunctiva, and cornea. Despite the similarities in the clinical picture, smallpox, and monkeypox differ primarily in that monkeypox causes lymph node enlargement (lymphadenopathy), whereas smallpox does not [78]. Moreover, in this current outbreak, atypical presentations are very commonly reported.

Monkeypox is usually a self-limiting disease. A person is contagious from the development of enanthem till the crusts fall off. Severe cases and worse outcomes commonly occur in children and individuals with underlying immune deficiencies.

### 5.4 Characteristic rash

**Fig. 6:**
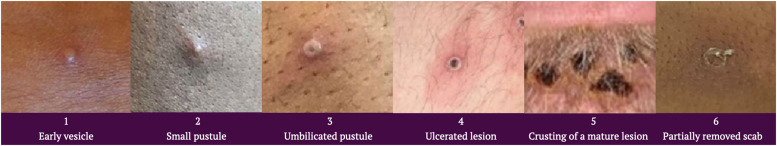
Evolution of the characteristic monkeypox skin rash. Courtesy of the UK Health Security Agency.

A study across 16 countries showed a characteristic rash in 95% of patients (the majority being in the anogenital region), with over 64% having more than 10 lesions present. The vesiculopustular lesions are deep and well-circumscribed with a characteristic umbilication with all lesions being of similar size, and at various stages across the site. Some cases of a single genital ulcer or lesion were also seen and warrant the possibility of misdiagnosis as a sexually transmitted infection [58]. This rash mostly presents as anogenital lesions while sparing the face and extremities or it may also be seen in the face, extremities, chest, and mucous membranes [81]. They are painful until they reach the final stages of crusting when they become itchy. The scab stage is thought to last around 14 days during which the lesions cycle through the following stages: enanthem (first lesions to develop on the tongue and mouth), macules (characterized by a flat base), papules (slightly raised firm lesions), vesicles (lesions filled with clear fluid), pustules (lesions filled with yellowish fluid), and crusts, which dry up and fall off [13,82] (Fig. 6).

### 5.5 Other characteristic symptoms

The current prodromal symptoms are seen to appear after the rash and have only been seen in around half of the cases, which again suggests the unusual nature of this 2022 outbreak [83]. Furthermore, patients with mucosal lesions presented with region-specific symptoms such as anorectal pain, proctitis, tenesmus, diarrhea with anorectal lesions and odynophagia, pharyngitis, and epiglottitis with oropharyngeal lesions. Possible complications include secondary infections, bronchopneumonia, and sepsis encephalitis [13,82].

### 5.6 Pediatric cases

Most cases of monkeypox currently seen in children are a result of household contact; however, the susceptibility and clinical outcomes are still unknown. Evidence shows increased severity in children younger than 8 years, children with underlying immune deficiencies, and children with pre-existing skin conditions. The presentation seen in children mimics the atypical pattern seen in adults with a characteristic rash that may or may not be accompanied by other symptoms. This rash may be misdiagnosed as other rashes commonly seen in children such as varicella, measles, scabies, allergic eruptions, etc. Complications are rare but include encephalitis, cellulitis, pneumonia, sepsis, abscess, and lymphadenopathy resulting in airway obstruction, keratitis, and corneal scarring [84].

## 6 Diagnosis

The factors that need to be considered in the diagnosis of monkeypox are the date of onset of fever and rash, recent travel history to endemic areas within 21 days preceding the onset of the symptoms, history of contact with a suspected infected person, date of specimen collection, and history of vaccination against smallpox. For clinical diagnosis, other rash illnesses like chickenpox, measles, bacterial skin infections, scabies, and syphilis must be excluded [13]. Collected samples are tested by PCR, which is considered the gold-standard diagnostic method owing to its accuracy and sensitivity. Diagnostic samples are collected from skin lesions, fluid from vesicles and pustules, and dry crusts. If available, biopsies can be employed to reach the diagnosis. Since orthopoxviruses are serologically cross-reactive, antigen and antibody detection methods are not specific to monkeypox. Moreover, a recent history of vaccination against smallpox may interfere with serological testing by giving false-positive results [13]. Testing for the presence of circulating anti-orthopoxvirus IgG and IgM can be done in individuals who have received a previous smallpox vaccination in which monkeypox infection is suspected. Moreover, rapid detection of active infection can be done using Tetracore Orthopox BioThreat Alert assay using samples from the patient's skin lesions. This assay, however, is nonspecific and can be used as a good negative test in endemic areas [20].

## 7 Clinical management

Infection from MPXV is milder as compared to smallpox and is usually self-limiting, resolving in about 2–4 weeks with bed rest and supportive care. Rarely, cases may develop severe dehydration, pitted and deforming scars, secondary bacterial infection, bronchopneumonia, respiratory distress, ocular infection, septicemia, and encephalitis. Management of the disease includes supportive treatment, antivirals, and immunoglobulins. Hospitalization in a negative pressure room is generally preferred for the management of complications [20].

### 7.1 Supportive care

The lack of effective antiviral drugs against MPXV, combined with the spontaneous recovery of most of the infected patients without the need for medical treatment [85], makes supportive and symptomatic therapy the mainstay of managing a monkeypox infection [24]. Table 3 provides insight into potential supportive care options that can be used to ease monkeypox symptoms [86].

### 7.2 Antiviral drugs

Cidofovir and brincidofovir (CMX-001) are antiviral agents with activity against *Orthopoxvirus* species. The former is an intravenously administered drug that inhibits viral DNA polymerases, whereas the latter is an orally administered modified cidofovir that has a better safety profile and is less nephrotoxic [20],[87]. A third drug, tecovirimat (aka TROXX and ST-246), approved under the US FDA's animal rule as an advanced therapy for the management of smallpox and licensed by the European Medical Association (EMA) for monkeypox in 2022, is an antiviral that inhibits the release of intracellular viruses [13,^20^,88].

For the current outbreak, tecovirimat and brincidofovir have been approved in the US to be used against monkeypox [42]. Moreover, the EMA licensed tecovirimat for monkeypox in 2022 [13,24], making it the only FDA and EMA-approved drug for *Orthopoxvirus* infection in humans [89].

*Tecovirimat* acts by inhibiting the VP37 viral envelope protein, which limits the last steps in viral maturation and release from the infected cell, preventing virus dissemination inside an infected host [90]. While the efficacy of this agent against monkeypox in humans has yet to be determined, studies in animals revealed that tecovirimat-treated animals outlived placebo-treated ones at various stages of the disease [88,91]. The drug is available in the form of an oral capsule or an intravenous vial [85].

*Brincidofovir* (oral) is a lipid conjugate of cidofovir, an acyclic nucleoside analog approved for the treatment of human cytomegalovirus retinitis in AIDS patients [42]. These agents work by inhibiting the viral DNA polymerase [92], and because brincidofovir can produce elevations in blood transaminases and bilirubin, liver function tests must be performed before and during treatment. Dual therapy with tecovirimat and brincidofovir may be used in patients with advanced disease [85]. However, it will not be ideal to use these drugs in all patients, reserving them to only patients that are at risk of developing, or people who are suffering from, severe disease [85].

## 8 Vaccines

Smallpox vaccines are at least 85% cross-protective against monkeypox. Pre-exposure prophylaxis against the virus can be achieved by various types of vaccines: a live-attenuated vaccinia vaccine (ACAM2000), a nonreplicating modified vaccinia Ankara (MVA), JYNNEOS, and a minimally replicating vaccine (LC16). Postexposure vaccination in unvaccinated individuals exposed to the disease effectively prevents future infections and, if given within 3 days of the exposure, can reduce the severity of the symptoms. JYNNEOS was shown to protect against the side effects documented in earlier vaccines. Nevertheless, the 28-day gap between the first and second dose may decrease its protective value in postexposure prophylaxis. Achieving mucosal immunity may play a pivotal role in preventing disease transmission, being mucous membranes a route by which the virus spreads. The ability of the available vaccines to produce mucosal immunity, however, is not yet confirmed [23].

### 8.1 Pre-exposure prophylaxis

Vaccinia Immune Globulin Intravenous (VIGIV) are antibodies obtained from the pooled blood of smallpox-vaccinated individuals. Although no clinical data is available on the efficacy of VIGIV for the management of monkeypox complications, it can be considered in combination with antivirals for severe infection. Moreover, the therapy is safe and may help treat vulnerable patients [89]. It is also FDA-approved for usage against monkeypox in the USA. Therefore, the CDC recommends immunocompromised individuals exposed to orthopoxviruses, including MPXV, be prophylactically given VIGIV to reduce the severity of infection [87].

Moreover, a study conducted by Stittelaar et al. concluded that “during an outbreak, cidofovir or other related nucleotide analogs should be considered as an additional strategy for vaccination of individuals exposed to smallpox or other poxviruses/orthopoxviruses, including monkeypox” [93]. Primary preventive vaccination is also recommended for MSM, healthcare workers, and laboratory workers who might be exposed to the virus. For these categories, prophylactic vaccination with ACAM2000, LC16, and the Modified Vaccinia Ankara–Bavarian Nordic (MVA-BN) vaccines are recommended [1].

### 8.2 Postexposure vaccination

MVA-BN vaccine, a third-generation live vaccine that is nonreplicable in humans [94], has lately been recommended for postexposure prophylaxis against monkeypox infection by several European and North American countries [94,95]. The evidence is driven by various animal studies [93,^96^,97]. Postexposure preventive immunization is recommended for high-risk groups like children, pregnant women, and individuals with immunosuppression. For this group of patients as well as breastfeeding women, minimally or nonreplicating vaccines such as MVA-BN and LC16 are recommended while replicating vaccines like ACAM2000 are contraindicated [1].

## 9 Public health measures

The key to preventing monkeypox is increasing public awareness of risk factors and minimizing exposure to viruses. According to the CDC's recommendations, any person exhibiting symptoms and signs should undergo complete investigations for confirmation of monkeypox. If confirmed, isolation and contact tracing are recommended to decrease the spread of the virus. Furthermore, contact with animals that might act as a reservoir of the virus should be avoided. In case of contact, hands should be immediately sanitized. According to WHO, vaccination against smallpox was demonstrated through several observational studies to be about 85% effective in preventing monkeypox [13]. Similarly, JYNNEOS (aka Imvamune or Imvanex) and ACAM2000 are 2 vaccines that have been approved by the US FDA for use in preventing monkeypox infection [98]. Monkeypox vaccination is being advised for close contacts (i.e., health workers) and further vaccine recommendations are being discussed by experts of the WHO [8]. Due to the cross-reactivity that the smallpox vaccine provides against monkeypox, the UK has distributed shots of the vaccines in an attempt to prevent the spreading outbreak [10]. During the most recent outbreak, Belgium was the first country to issue a 21-day quarantine for patients who have tested positive for MPXV after confirming its third case [99]. The US, contrastingly, confirmed that quarantine will not be mandatory due to its surplus of smallpox vaccines enough to prevent any serious outbreak [100].

## 10 Infection control measures

### 10.1 When isolating patients at home

Monkeypox patients should be isolated at home until the rash has completely disappeared. This is because infection from the nonintact skin is highest in monkeypox. Having visitations, close contact with people and animals, or sexual activity are high-risk behaviors that can lead to the rapid transmission of the disease. Patients should not share any materials (e.g., plates, cutlery, clothing, towels) with other household members. A separate bathroom is favorable, but if that is not feasible, disinfecting the bathroom after usage with alcohol-based materials should be done. Proper hand hygiene and wearing gloves and face masks should be exercised regularly if contact is imminent. The CDC also recommends wearing tall clothes covering most of the body area [101].

### 10.2 When handling patients in hospitals and clinics

Because monkeypox is mostly spread through physical contact, the following activities are advised in hospitals and clinics besides standard precautions applied to all patients. Monkeypox patients should adhere to transmission-based precautions, specifically contact precautions, which include isolating them in a separate room, cleaning and disinfecting the room at least daily, performing proper hand hygiene techniques with soap and water or using an alcohol-based hand sanitizer, limiting transport and movement of patients outside the room unless for medically-necessary purposes, and providing individual patient care equipment such as stethoscopes and blood pressure cuffs. Healthcare workers should use personal protective equipment (PPE) before entering the patient's room. This typically includes wearing gloves, gown, eye protection, and NIOSH-approved particulate respiratory equipped with at least N95 filters for all interactions. Removing PPE before leaving the room, performing a hand hygiene routine immediately, and ensuring that no surfaces or objects in the patient's room are touched after the removal of PPE are also required measures to ensure the safety of healthcare personnel and other patients in the hospital [102].

### 10.3 When handling specimens from patients

The amount of pox virus that is expected to be present in clinical samples of blood and bodily fluids is minimal. Because of this, immunization is not advised for staff who handle and analyze common clinical specimens from monkeypox patients (such as urine for urinalysis, blood for complete blood count (CBC), chemistries, and microbiology). It is advised to use biosafety level 2 (BSL-2) confinement to take general precautions to safeguard against any potential infectious pathogens present in any specimen received. The MPXV will not be present in clinical specimens if standard precautions and biosafety measures are consistently followed for the protection of laboratory personnel. Specimen testing should only be performed by a small number of employees, and processes that could produce infectious aerosols should be avoided [103].

### 10.4 Waste and environmental management

All wastes collected from the patient should be promptly inactivated on-site or safely sent to a special facility that is specialized in dealing with Category A waste of infectious substances, according to the US Department of Transportation (DOT) [104]. These measures include autoclaving, chemical disinfection, or the usage of alkaline hydrolysis digesters. Laundry (e.g., clothes, beddings) obtained from the patient should be placed in a laundry bag using PPE and with caution not to shake the materials. Cleaning activities that can lead to the dispersion of molecules (e.g., sweeping) should be replaced with wet cleaning methods [102].

### 10.5 Surveillance and containment

The main goals of surveillance and case investigation for monkeypox are to quickly identify cases, clusters, and the sources of infection to provide the best clinical care, isolate cases to stop further transmission, identify and manage contacts, and develop efficient control and prevention strategies based on the most frequently discovered routes of transmission. According to the WHO, the definition of contact has had one more of the following exposures with a probable or confirmed case of monkeypox: face-to-face exposure (including health care workers without appropriate PPE), direct physical contact, including sexual contact, contact with contaminated materials such as clothing or bedding. For 21 days after the last contact with a patient or their contaminated materials during the infectious period, contacts should be checked at least once a day for the appearance of signs or symptoms [9]. At the same time, people with a history of travel from countries with a high number of cases are also encouraged to self-monitor for any symptoms. All foods containing animal meat or parts must be thoroughly cooked before eating, and restrictions on animal trade are measures to prevent zoonotic transmission [13].

## 11 Working through health inequalities

During the most recent outbreak, it has been revealed that the virus was spreading undetected in various African countries where multiple outbreaks have happened before. However, before the spread to the Western world, there were no statistics on how serious the problem was, except for the report published by the WHO, stating that Cameroon, Central African Republic, Congo, and Nigeria have reported several monkeypox cases (28, 17, 7, and 110, respectively) and deaths (22, 3, and 1 respectively) in early 2022. Furthermore, the DRC has experienced a surge in the number of monkeypox cases since March 2019. Surprisingly, the investigation for this outbreak started 21 months later (January 2021), and that is when the public health team identified 1,238 cases and 57 deaths in 2022 [105]. It, therefore, appears that a severe outbreak was taking place in DRC, whereas in other endemic countries, the virus has been spreading with its normal background incidence.

## 12 Conclusions

This review serves to provide a comprehensive understanding of MPXV virology, genomic structure, transmission, diagnosis, management, and infection control measures. Despite the numerous unknowns about the recent monkeypox outbreak, its mild clinical picture makes it easier to treat. Still, implementing wide public health measures is important to hinder the spread of the virus, although its potential to progress into a pandemic is unlikely. Although the certain reason behind the sudden re-emergence of the virus is unknown, one of the strongest hypotheses is the decrease in smallpox vaccination coverage following its eradication. Nevertheless, haphazardly injecting the populations with smallpox or monkeypox vaccines should be discouraged. The fear of a “new COVID-19” shall not hinder our scientific judgment in dealing with future monkeypox outbreaks the right way and care should be taken in avoiding the shortcomings that happened during the COVID-19 outbreak. A thorough investigation of the reasons that led to the virus going undetected for a long time in LMICs is paramount in tackling the threat of future outbreaks. Finally, the adoption of a One Health approach for the timely detection and response to re-emerging zoonotic infections is essential.

## CRediT authorship contribution statement

R.E.: Conceptualization, Data curation, Visualization, Methodology, Project administration, Resources, Writing – original draft. A.M.M.: Conceptualization, Data curation, Visualization, Methodology, Project administration, Resources, Writing – original draft. T.V.: Conceptualization, Writing – original draft. S.T.: Writing – original draft. K.S.: Writing – original draft. N.D.T.: Writing – original draft. S.S.E.: Writing – original draft. N.T.V.: Writing – original draft. N.T.H.: Conceptualization, Data curation, Project administration.
